# Optimal search strategies for identifying mental health content in MEDLINE: an analytic survey

**DOI:** 10.1186/1744-859X-5-4

**Published:** 2006-03-23

**Authors:** Nancy L Wilczynski, R Brian Haynes, Team Hedges

**Affiliations:** 1 Department of Clinical Epidemiology and Biostatistics, McMaster University, Health Sciences Centre, 1200 Main Street West, Hamilton, Ontario, L8N 3Z5, Canada; 2 Department of Medicine, McMaster University, Michael G. DeGroote School of Medicine, Health Sciences Centre, 1200 Main Street West, Hamilton, Ontario, L8N 3Z5, Canada

## Abstract

**Objective:**

General practitioners, mental health practitioners, and researchers wishing to retrieve the best current research evidence in the content area of mental health may have a difficult time when searching large electronic databases such as MEDLINE. When MEDLINE is searched unaided, key articles are often missed while retrieving many articles that are irrelevant to the search. The objectives of this study were to develop optimal search strategies to detect articles with mental health content and to determine the effect of combining mental health content search strategies with methodologic search strategies calibrated to detect the best studies of treatment.

**Method:**

An analytic survey was conducted, comparing hand searches of 29 journals with retrievals from MEDLINE for 3,395 candidate search terms and 11,317 combinations. The sensitivity, specificity, precision, and accuracy of the search strategies were calculated.

**Results:**

3,277 (26.8%) of the 12,233 articles classified in the 29 journals were considered to be of interest to the discipline area of mental health. Search term combinations reached peak sensitivities of 98.4% with specificity at 50.0%, whereas combinations of search terms to optimize specificity reached peak specificities of 97.1% with sensitivity at 51.7%. Combining content search strategies with methodologic search strategies for treatment led to improved precision: substantive decreases in the number of articles that needed to be sorted through in order to find target articles.

**Conclusion:**

Empirically derived search strategies can achieve high sensitivity and specificity for retrieving mental health content from MEDLINE. Combining content search strategies with methodologic search strategies led to more precise searches.

## Background

Retrieving the best current evidence for a specific medical discipline when searching in large electronic databases such as MEDLINE can be challenging. This challenge is due to the scatter of relevant articles in low concentration across a large number of journals, inherent limits in indexing, and lack of searching skill on the part of the user of the database [1]. For instance, MEDLINE searches take place in a database containing over 13 million citations from over 4,800 journals with over 571,000 new articles added each year [2]. MEDLINE includes articles on basic biomedical research and the clinical sciences including nursing, dentistry, veterinary medicine, pharmacy, allied health, and pre-clinical sciences and also covers life sciences, including some aspects of biology, environmental science, marine biology, plant and animal science as well as biophysics and chemistry [2]. Attempting to find articles relevant to a specific area or topic can be daunting for the searcher.

Researchers have developed search strategies to help retrieve scientifically sound, clinically relevant articles while searching in MEDLINE. To date the majority of the search strategies have been developed when searching for therapy, diagnostic and review articles [3-13]. In addition to these areas, we have also developed search strategies to identify scientifically sound, clinically relevant articles about causation, prognosis, economics, clinical prediction, and studies of a qualitative nature [14-21]. These search strategies have been adapted for use in the Clinical Queries interface of PubMed  as well as the limits screen of Ovid .

Although these search strategies are helpful in identifying scientifically sound, clinically relevant articles for clinical matters (e.g., treatment), they are not designed to detect content for any particular disorder (e.g., depression). When conducting a "usual" search in MEDLINE, content terms would be "ANDed" to the methodologic search strategies that have been developed (e.g., diabetes mellitus, type I.sh. AND randomized controlled trial.mp,pt.). To date, we are unaware of any studies reporting empirically tested search strategies for identifying articles for a particular disease or clinical discipline combined with methodologic search terms.

The objectives of this study were to develop optimal search strategies to detect articles of interest to the discipline area of mental health and to determine the effect that content search strategies have on the performance of methodologic search strategies for treatment when the strategies are combined using the Boolean "AND".

## Methods

We compared the retrieval performance of mental health content search terms in MEDLINE with a manual review (hand search) of each article for each issue of 29 journal titles for the year 2000. Overall research staff hand searched 170 journal titles. These journals were chosen based on recommendations of clinicians and librarians, Science Citation Index Impact Factors provided by the Institute for Scientific Information, and ongoing assessment of their yield of studies and reviews of scientific merit and clinical relevance for the disciplines of internal medicine, general medical practice, mental health, and general nursing practice (list of journals provided by the authors upon request). Of these 170 hand searched journals, 161 were indexed in MEDLINE. Search strategies for the study we report here were developed using a 29 journal-subset chosen based on those journals that had the highest number of methodologically sound studies in the area of mental health, that is, those that contributed > 1 article to the journal *Evidence-Based Mental Health * during the year 2000 (list of journals provided by the authors upon request).

We compiled a list of 3,395 index terms and textwords (list of terms tested provided by the authors upon request). This list was compiled after surveying 140 mental health specialists from around the world, reviewing the search strategies from 5 mental health focused Cochrane groups, and mapping textwords to MeSH terms. Examples of the search terms tested are '(learn: adj problem)', 'schizoid', 'depression', and 'mania', all as textwords; 'phobic disorders', the index term; and the index term 'aggression', exploded (i.e., a search term that automatically includes closely related indexing terms).

As part of a larger study [22], 6 trained, experienced research assistants read all issues of 170 journals for the publishing year 2000. Each article was rated using purpose and quality indicators and categorized into clinically relevant original studies, review articles, general papers, or case reports. The original and review articles were then categorized as 'pass' or 'fail' for methodologic rigor in the areas of therapy/quality improvement, diagnosis, prognosis, causation, economics, clinical prediction, and review articles. The research staff were rigorously calibrated before reviewing the journals and inter-rater agreement for identifying the format of articles (e.g., original study, review article) was 92% beyond chance (kappa statistic, 95% confidence interval (CI) 0.89 to 0.95). Inter-rater agreement for which articles met all scientific criteria (e.g., treatment study, diagnostic study) was 89% beyond chance (kappa statistic, CI 0.78 to 0.99) [22]. One research assistant then hand searched all articles in each issue of the 29 journal subset and indicated if the article was of interest to the area of mental health. The predetermined criteria for "of interest to mental health" were as follows:

Pharmacological interventions for persons with mental health problems; cognitive and behavorial approaches to helping any patient (e.g., including cancer patients); etiology pertaining to mental health; diagnosis pertaining to mental health; or economic issues pertaining to mental health.

The proposed search strategies were treated as "diagnostic tests" for sound studies and the manual review (hand search) of the literature was treated as the "gold standard". We determined the sensitivity, specificity, precision, and accuracy of each single term and combinations of terms in MEDLINE using an automated process. Sensitivity for a given topic is defined as the proportion of high quality articles for that topic that are retrieved; specificity is the proportion of low quality articles not retrieved; precision is the proportion of retrieved articles that are of high quality; and accuracy is the proportion of all articles that are correctly classified.

Individual search terms with sensitivity > 15% and specificity > 80% for articles of interest to mental health were incorporated into the development of search strategies that included 2 or more terms. All combinations of terms used the Boolean OR, for example, "mania.tw. OR depression.sh.". For the development of multiple-term search strategies to optimize either sensitivity or specificity, we tested all 2-term search strategies with sensitivity at least 75% and specificity at least 50%. For optimizing accuracy, 2-term search strategies with accuracy > 75% were considered for multiple-term development. 11,317 search strategies were tested in the development of mental health content search filters. To enhance the performance of the most sensitive mental health content search strategy, the single search terms with the highest sensitivity were successively added to the top performing 3-term search strategy until the best sensitivity was achieved while keeping specificity ≥50%.

In addition to developing mental heath content search strategies as just described, we also evaluated the performance of the methodologic search filters for treatment articles when "ANDed" with the mental health content filters.

## Results

Indexing information was downloaded from MEDLINE for 12,233 articles from the 29 journals hand searched. Of these 3,277 (26.8%) were considered to be of interest to mental health. Search strategies were developed using all 12,233 articles. Thus, the strategies were tested for their ability to retrieve mental health articles from all other articles.

Table 1 shows the best single term for high-sensitivity, high-specificity, and best balance of sensitivity and specificity. The single term, exp mental disorders, produced the best sensitivity of 74.7% while keeping specificity at 94.0%. This term also produced the highest specificity and the optimal balance between sensitivity and specificity.

**Table 1:** Single term with the best sensitivity (keeping specificity ≥50%), best specificity (keeping sensitivity ≥50%), and best optimization of sensitivity and specificity (based on the lowest possible absolute difference between sensitivity and specificity) for detecting mental health content in MEDLINE in 2000

Search term OVID search*	**Sensitivity (%) (95% CI)** ** n = 3,277**	**Specificity (%) (95% CI)** ** n = 8,956**	**Precision (%) (95% CI)** ** n = 2,990**	**Accuracy (%) (95% CI)** ** n = 12,233**
**Best sensitivity, best specificity and best optimization of sensitivity & specificity**				
exp mental disorders	74.7 (73.3 to 76.2)	94.0 (93.5 to 94.5)	81.1 (80.5 to 83.3)	88.8 (88.3 to 89.4)

*Search strategies are reported using Ovid's search engine syntax for MEDLINE.

exp = explode, a search term that automatically includes closely related indexing terms.

Combination of terms with the best results for sensitivity, specificity and optimization of sensitivity and specificity are shown in Tables 2, 3, 4. Combinations of terms improved on single search term performance for sensitivity. The 29-term search strategy shown in Table 2 achieved a sensitivity of 98.4% (a 23.7% improvement over the single term) while keeping specificity at 50.0%. The 3-term strategy shown in Table 3, psychiatr:.mp., OR exp mood disorders OR psycho:.tw., had the highest specificity at 97.1% (a 3.1% increase over the single term) while keeping sensitivity at 51.7%. The 4-term combination shown in Table 4, depress:.mp. OR behav:.mp. OR exp mental disorders OR psych:.mp., resulted in the best optimization strategy achieving above 89% for both sensitivity and specificity.

**Table 2:** Combination of terms with the best sensitivity (keeping specificity ≥50%) for detecting mental health content in MEDLINE in 2000 and performance when combined with the most sensitive strategy for detecting treatment studies

Search Strategy OVID search*	**Sensitivity (%) (95% CI)**	**Specificity (%) (95% CI)**	**Precision (%) (95% CI)**	**Accuracy (%) (95% CI)**
**Best Sensitivity**				
depress:.mp. OR adolescen:.mp. OR exp mental disorders OR psych:.mp. OR "use disorder:".tw. OR behav:.mp. OR exp psychotropic drugs OR exp psychology, social OR neuro:.mp. OR dt.fs. OR exp brain diseases OR cognitive:.mp. OR exp neurotransmitter agents OR exp psychotherapy OR exp social problems OR anxiety.mp. OR attention:.mp. OR exp emotions OR exp neurobehavioral manifestations OR chronic.tw. OR mental health.mp. OR stress.mp. OR alcohol.mp. OR abus:.mp. OR prevent:.mp. OR stress, psychological.sh. OR exp adaptation, psychological OR outcome measure:.tw. OR exp mental health services	(n = 3,277) 98.4 (98.0 to 98.9)	(n = 8,956) 50.0 (49.0 to 51.1)	(n = 7,700) 41.9 (40.8 to 43.0)	(n = 12,233) 63.0 (62.2 to 63.9)

**Above strategy "ANDed" with best sensitivity strategy for detecting methodology sound treatment studies**				
clinical trial.mp. OR clinical trial.pt. OR random:.mp. OR tu.xs.	(n = 129) 99.2 (95.8 to 100.0)	(n = 7,571) 69.8 (68.8 to 70.8)	(n = 2,414) 5.3 (4.4 to 6.3)	(n = 7,700) 70.3 (69.3 to 71.3)

*Search strategies are reported using Ovid's search engine syntax for MEDLINE.

: = truncation; mp = multiple posting – term appears in title, abstract, or subject heading; exp = explode, a search term that automatically includes closely related indexing terms; tw = textword (word or phrase appears in title or abstract); dt = drug therapy; fs = floating subheading; sh = MeSH, medical subject heading; pt = publication type; tu = therapeutic use; xs = exploded subheading.

**Table 3:** Combination of terms with the best specificity (keeping sensitivity ≥50%) for detecting mental health content in MEDLINE in 2000 and performance when combined with the most specific strategy for detecting treatment studies

Search Strategy OVID search*	**Sensitivity (%) (95% CI)**	**Specificity (%) (95% CI)**	**Precision (%) (95% CI)**	**Accuracy (%) (95% CI)**
**Best Specificity**				
psychiatr:.mp. OR exp mood disorders OR psycho:.tw.	(n = 3,277) 51.7 (50.0 to 53.4)	(n = 8,956) 97.1 (96.7 to 97.4)	(n = 1,954) 86.6 (85.1 to 88.2)	(n = 12,233) 84.9 (84.3 to 85.6)

**Above strategy "ANDed" with best specificity strategy for detecting methodology sound treatment studies**				
randomized controlled trial.mp. OR randomized controlled trial.pt.	(n = 129) 62.0 (53.1 to 70.4)	(n = 1,825) 98.0 (97.2 to 98.6)	(n = 117) 68.4 (59.1 to 76.7)	(n = 1,954) 95.6 (94.6 to 96.5)

*Search strategies are reported using Ovid's search engine syntax for MEDLINE.

: = truncation; mp = multiple posting – term appears in title, abstract, or subject heading; exp = explode, a search term that automatically includes closely related indexing terms; tw = textword (word or phrase appears in title or abstract); pt = publication type.

**Table 4:** Combination of terms with the best optimization of sensitivity and specificity (based on the lowest possible absolute difference between sensitivity and specificity) for detecting mental health content in MEDLINE in 2000 and performance when combined with the best optimization strategy for detecting treatment studies

Search Strategy OVID search*	**Sensitivity (%) (95% CI)**	**Specificity (%) (95% CI)**	**Precision (%) (95% CI)**	**Accuracy (%) (95% CI)**
**Best Optimization of Sensitivity and Specificity**				
depress:.mp. OR behav:.mp. OR exp mental disorders OR psych:.mp.	(n = 3,277) 89.2 (88.1 to 90.3)	(n = 8,956) 89.7 (89.1 to 90.4)	(n = 3,844) 76.0 (74.7 to 77.4)	(n = 12,233) 89.6 (89.0 to 90.1)

**Above strategy "ANDed" with best optimization strategy for detecting methodology sound treatment studies**				
randomized controlled trial.pt. OR randomized.mp. OR placebo.mp.	(n = 129) 92.3 (87.6 to 96.9)	(n = 3,715) 95.0 (94.2 to 95.7)	(n = 304) 39.0 (33.5 to 44.7)	(n = 3,844) 94.9 (94.2 to 95.6)

*Search strategies are reported using Ovid's search engine syntax for MEDLINE.

: = truncation; mp = multiple posting – term appears in title, abstract, or subject heading; exp = explode, a search term that automatically includes closely related indexing terms; pt = publication type.

Each of the top performing strategies for detecting mental health content were "ANDed" with the top performing methodologic search strategies for detecting scientifically sound, clinically relevant treatment studies. The results of these combinations are also shown in Tables 2, 3, 4. Comparing the search results of the most sensitive mental health content strategy alone with the results when it was combined with the most sensitive methodologic treatment strategy we found a 3-fold decrease in the absolute number of articles to be sorted through to detect those articles on target, that is, those articles with mental health content that were scientifically sound and clinically relevant for evaluating a treatment question (Table 2; 7,700 vs. 2,414). This means that when searching for scientifically sound treatment articles on mental health topics using the mental health content search strategy alone 1.7% of the retrieved articles were on target (1 out of every 60 articles). However, when searching for scientifically sound treatment articles on mental health topics using the mental health content search strategy combined with the most sensitive methodologic treatment strategy 5.3% of the retrieved articles were on target (1 out of every 19 articles). This effect was more dramatic when searching using the most specific strategies: a 17-fold absolute decrease was found (Table 3; 1,954 [1 out of every 29 articles were on target] vs. 117 [1 out of every 1.5]) whereas when using the optimization strategies, there was a 13-fold decrease (Table 4; 3,844 [1 out of every 33 articles were on target] vs. 304 [1 out of every 2.5]). Although there was a gain in terms of having to shift through fewer articles to find one on target, these search strategies do lead to some loses. For instance, when searching using the most sensitive combination just one on target article was lost. This loss is small because the sensitivity is so high. However, when searching using the most specific combination that loss was more substantive, 40 on-target articles were lost. The optimal combination led to 10 on target articles being missed.

## Discussion

Our study documents search strategies that can help discriminate the literature with mental health content from articles that do not have mental health content. General practitioners, mental health practitioners, and researchers wanting an overview of the best current evidence in the area of mental health will best be served by the most sensitive search strategy when they have time to sort through articles. This search will have the highest probability of retrieving all relevant articles (in this study one on-target article missed), but will have the lowest precision, retrieving many irrelevant articles. With less time on their hands general practitioners, mental health practitioners, and researchers they may wish to search with the strategy that optimizes the balance between sensitivity and specificity (10 on target articles missed) or the strategy that optimizes specificity (40 on target articles missed).

As indicated in our previous papers [14-21], when searching with the methodologic search filters alone we found that precision was generally low and therefore of concern. This was expected given the low proportion of relevant target articles for a given purpose in a very large, multipurpose database. This means that searchers will continue to need to spend time discarding irrelevant retrievals.

As reported in this paper, we set out to test whether precision would be enhanced by combining the methodologic search strategies with content specific terms using the Boolean 'AND'. We found a 3- to 17-fold decrease in the absolute number of articles that would need to be sorted through to find articles that are on target. This decrease is substantive and shows that combining empirically derived search strategies for enhancing the retrieval of relevant content with search strategies derived for enhancing the retrieval of scientifically sound, clinically relevant articles can have a profound impact on searching.

The example used in this paper is for retrieving high quality treatment papers with mental health content. Treatment was used because the sample size was sufficient to test the performance of combined search strategies (content and methods) in this 29 journal subset (n = 129). Other purpose categories, for example diagnosis, did not lend themselves to this test because the number of scientifically sound diagnostic articles with mental health content in this 29 journal subset was low (e.g., pass diagnosis articles with mental health content, n = 29).

## Conclusion

Selected combinations of indexing terms and textwords can achieve high sensitivity or specificity in retrieving articles with mental health content in MEDLINE. Combining content search strategies with methodologic search strategies can lead to a substantive decrease in the absolute number of articles that need to be sorted through to find those articles that are on target.

## Competing interests

The author(s) declare that they have no competing interests.

## Conflict of interest statement

No conflicts of interest. Both authors, Nancy L. Wilczynski and R. Brian Haynes, had full access to all the data in the study and had final responsibility for the decision to submit for publication.

## Authors' contributions

RBH and NLW prepared grant submissions in relation to this project. Both authors drafted, commented on and approved the final manuscript. Both authors also supplied intellectual content to the collection and analysis of the data. NLW participated in the data collection and both authors were involved in data analysis and staff supervision.
